# Efficacy and safety of berberine in the treatment of type 2 diabetes with insulin resistance: Protocol for a systematic review

**DOI:** 10.1097/MD.0000000000016947

**Published:** 2019-08-30

**Authors:** Yinshan Wang, Aihua Yan, Shanshan Li, Bei Liu, Huimin Li, Yong Yan

**Affiliations:** a Department of Endocrinology, Kaifeng Hospital of Traditional Chinese Medicine, Kaifeng; b Henan University of Chinese Medicine, Zhengzhou, China.

**Keywords:** berberine, insulin resistance, protocol, systematic review, type 2 diabetes mellitus

## Abstract

**Background:**

The incidence of diabetes mellitus (DM) is increasing year by year, and various complications can endanger the lives of patients. Type 2 diabetes mellitus (T2DM) accounts for more than 90% of DM, most of which is associated with insulin resistance (IR), and IR has been shown to be closely related to the onset of T2DM and the presence of DM complications. Berberine (BBR) has been shown to improve T2DM with IR in a number of ways. In this study, we aim to evaluate the efficacy and safety of BBR in the treatment of T2DM with IR to provide the newest evidence for clinical use.

**Methods and analysis:**

Literature research will be divided into 2 parts: electronic search and manual search. We will search PubMed, EMBASE, The Cochrane Library, the China National Knowledge Infrastructure, China Biology Medicine disc, the China Science and Technology Journal database, and the Wanfang database online. We will select the eligible studies published up to June 30, 2019. Dissertations, conference papers, ongoing trials, internal reports, etc., are searched by manual search methods. We use Homeostatic Model Assessment for IR (HOMA-IR) as the primary outcome of T2DM with IR, and we will also focus on the patient's blood glucose levels and all adverse reactions that occur during medication.

Two reviewers will read the articles, extract the data information, and assess the risk of bias independently. Data analysis will use the software such as RevMan 5.3.5, ENDNOTE X7, and STATA 13.0.

**Results:**

This study will provide a high-quality synthesis of current evidence of BBR for T2DM with IR from several aspects including HOMA-IR, blood glucose levels, and adverse events.

**Conclusion:**

This systematic review will provide evidence to assess the efficacy and safety of BBR in the treatment of T2DM with IR.

**Ethics and dissemination:**

Because all of the data used in this systematic review has been published, ethical approval is not required.

**Trial registration number:**

PROSPERO CRD42019123225.

## 1 Introduction

Diabetes mellitus (DM) is a common metabolic disease, its incidence is increasing year by year, and various complications seriously affect the quality of life of patients and are even life-threatening. Adult diabetes is expected to reach 439 million by 2030, accounting for 7.7% of the adult population.^[1,2]^

Type 2 diabetes mellitus (T2DM) specifically refers to the glucose and fat metabolic disorder syndrome caused by insulin resistance (IR) and insufficient insulin secretion, accounting for more than 90% of the total number of diabetes.^[3,4]^ IR refers to the decreased sensitivity of insulin to peripheral target tissues such as liver, muscle, and adipose tissue, insulin-induced glucose uptake and utilization efficiency, resulting in a series of clinical manifestations such as hyperglycemia, hyperinsulinemia, and dyslipidemia. IR is also considered to be a contributing factor to T2DM.^[5]^ The development of effective, low-toxic drugs for IR is important for the control of T2DM.

For the treatment, the Homeostatic Model Assessment for IR (HOMA-IR) is used as a standard for evaluating the degree of IR.^[6,7]^ The drugs that improve T2DM with IR mainly include sulfonylureas,^[8]^ biguanides,^[9]^ Alpha-glucosidase inhibitors,^[10]^ thiazolidinediones,^[11]^ but are often accompanied by many adverse reactions such as edema, cardiovascular disease, and liver and kidney dysfunction. Berberine (BBR) is the main component of Coptis, with low toxicity and low cost. At present, the role of berberine in regulating and improving glycolipid metabolism has been confirmed.^[12–14]^ There are also many studies showing that BBR can effectively improve T2DM with IR,^[15–17]^ but it lacks relevant meta-analysis. This study aimed to systematically evaluate the efficacy and safety of BBR treatment with T2DM with IR using a meta-analysis method to provide the best evidence for its clinical application.

## 2 Methods

The protocol has been registered on PROSPERO as CRD42019123225 (https://www.crd.york.ac.uk/prospero/display_record.php?RecordID=123225). The protocol is written in accordance with the Systematic Review and Meta-analysis Program (PRISMA-P) Statement. If necessary, we will describe the changes in the full review.

### 2.1 Inclusion criteria for study selection

#### 2.1.1 Types of studies

Randomized controlled trials (RCTs) of BBR for the treatment of T2DM with IR will be included in the research, with language limited to Chinese and English. Observational studies, nonrandomized controlled studies, case report will be excluded.

#### 2.1.2 Types of participants

Patients diagnosed with T2DM with IR will be included, while age, gender, region, ethnicity, and source are not restricted.

#### 2.1.3 Types of interventions

The treatment group will use the BBR, with no limitation of the dose and frequency of the medicine. At the same time, the control group will use placebo for intervention. The trial period requires more than 1 course of treatment.

#### 2.1.4 Types of outcome measures

##### 2.1.4.1 Primary outcomes

We use HOMA-IR as the primary outcome of T2DM with IR.

##### 2.1.4.2 Secondary outcomes

We also focus on the patient's blood glucose levels and all adverse reactions that occur during medication.

### 2.2 Search methods for the identification of studies

#### 2.2.1 Electronic searches

Literature research will be divided into 2 parts: electronic search and manual search. We will search PubMed, EMBASE, The Cochrane Library, the China National Knowledge Infrastructure, China Biology Medicine disc, the China Science and Technology Journal database, and the Wanfang database online. We will select the eligible studies published up to March 31, 2019. The search terms used in the systematic review are as follows: Berberine, BBR, Type 2 diabetes mellitus with insulin resistance, Type 2 diabetes mellitus, insulin resistance, T2DM with IR, T2DM, and IR.

The specific search strategy will be (taking PubMed as an example):

Search ((((Type 2 diabetes mellitus with insulin resistance [Title/Abstract] OR Type 2 diabetes mellitus [Title/Abstract] OR Insulin resistance [Title/Abstract] OR T2DM with IR [Title/Abstract] OR T2DM [Title/Abstract] OR IR [Title/Abstract]))) AND (Berberine [Title/Abstract] OR BBR[Title/Abstract]))

And a similar search strategy will be applied to other electronic databases.

#### 2.2.2 Searching other resources

Dissertations, conference papers, ongoing trials, internal reports, etc., are searched by manual search methods. At the same time, we will also retrieve ongoing trials related to T2DM with IR from the clinical registration platform, such as the WHO International Clinical Trial Registry Platform. And we will also try to contact the test leader for further information if necessary.

### 2.3 Data collection and analysis

#### 2.3.1 Selection of studies

**Figure 1:**
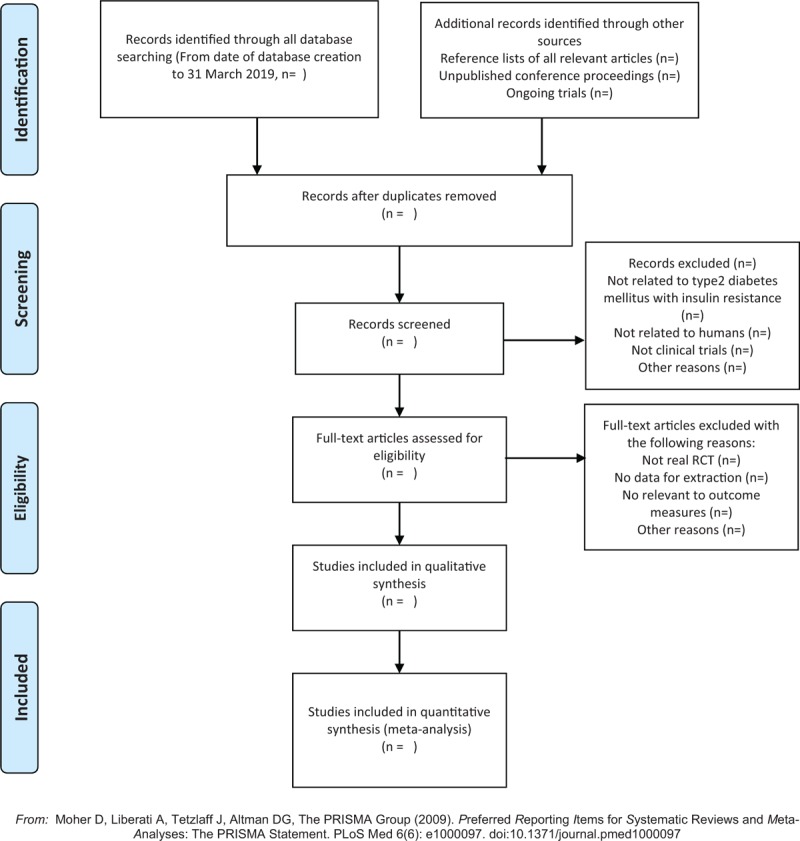
The PRISMA flow chart. PRISMA = preferred reporting items for systematic reviews and meta-analyses.

First, 2 reviewers will independently read the titles of the references to exclude the obviously irrelevant literatures, and then they will read the abstract and full text to determine whether the studies would be finally included. EndNote X7 literature management software will be used for the screening process. And the reviewers will contact the author for the complete information if needed. If there is a disagreement, another reviewer will decide it. And we will also record the exclusion reason for the excluded literature. The details of selection process will be shown in the PRISMA flow chart (Fig. 1).

#### 2.3.2 Data extraction and management

Two reviewers will retrieve the following data independently: study details (authors, country, year of publication, multicenter study, or not), participant details (baseline data, diagnostic criteria), the methods used (sample size, blinding method), the interventions used in both treatment and control group, the primary and secondary outcomes (HOMA-IR, blood glucose levels, adverse events). And we will contact the corresponding authors for the data mentioned above if the data is incomplete. And if there is a disagreement, all reviewers will join the discussion to deal with it.

#### 2.3.3 Assessment of risk of bias in included studies

Two reviewers will assess the quality of each article independently using the risk of bias (ROB) assessment tool in the Cochrane. Random sequence generation, allocation concealment, blindness assessment of results, incomplete outcome data, selective reporting, and other biases will be all evaluated. The results will be: low-risk, unclear, and high-risk. If there is a disagreement, a group discussion will be conducted to make a decision.

#### 2.3.4 Measures of treatment effect

For continuous variable outcomes, we will record the mean difference (MD) or standardized mean difference (SMD) and 95% confidence interval (CI). And we will record the relative risk (RR) and 95% CI for dichotomous outcomes.

#### 2.3.5 Dealing with missing data

If the information of the article is missing, we will contact the author for further information. If the necessary information is not obtained, we will use the available data for data synthesis. And we will also discuss the possible consequences of missing data.

#### 2.3.6 Assessment of heterogeneity

*I*^2^ will be used to determine the heterogeneity. When *I*^2^ ≤ 50%, heterogeneity is considered acceptable. And when *I*^2^ > 50%, the heterogeneity among the trials will be considered significant, and further subgroup analysis would be conducted to identify possible causes.

#### 2.3.7 Data synthesis and analysis

We will use Review Manager software (RevMan V.5.3.5) provided by Cochrane

Collaboration for data synthesis and analysis. When *I*^2^ ≤ 50%, a fixed-effects model will be used to calculate the RR and MD; on the contrary, we will use a random-effects model. If the heterogeneity is significant, we will analyze the cause of the heterogeneity by subgroup analysis or meta regression analysis.

#### 2.3.8 Assessment of publication bias

We will use a funnel plot to judge whether a publication bias exists when at least 10 trials are included in the research. And when the number is less than 10, the publication bias assessment will be determined by STATA 13.0.

#### 2.3.9 Subgroup analysis

Subgroup analysis is to explore the source of heterogeneity. When more than 10 studies are included, subgroup analyses can be performed according to interventions, participants, age, gender, duration of disease, and dose.

#### 2.3.10 Sensitivity analysis

Sensitivity analysis will be performed to examine the robustness of conclusions. We will re-analyze whether the conclusions have changed by determining the effects of excluding studies with high risks of bias, studies with missing data, and outliers.

#### 2.3.11 Grading the quality of evidence

It is recommended to use the Grading of Recommendations Assessment, Development and Evaluation (GRADE)^[18]^ to analyze the quality level of evidence. Bias risks; heterogeneity; publication bias and other factors will be considered. The quality of the evidence will be graded using “very low,” “low,” “moderate,” or “high.”

## 3 Discussion

IR is the main mechanism of T2DM pathogenesis and runs through the whole process of T2DM, and related studies have confirmed that IR is closely related to the progress of complications of DM.^[19,20]^

**Figure 2:**
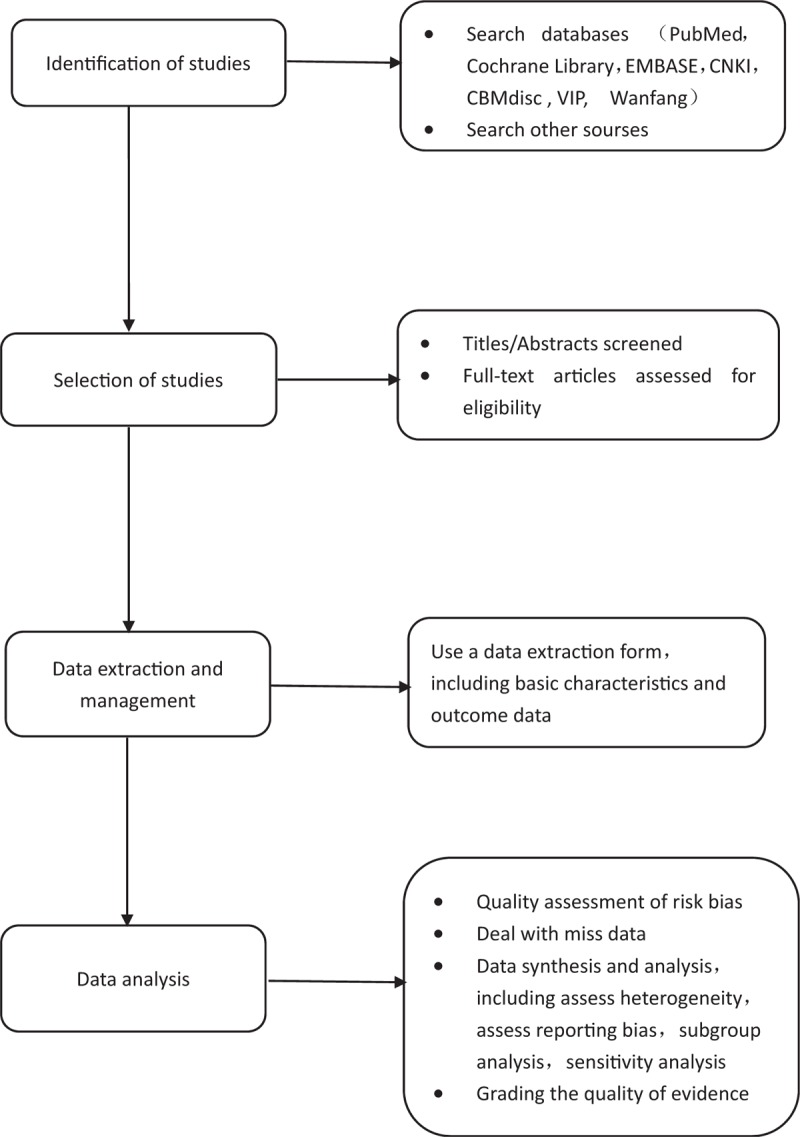
Flow diagram of the systematic review and meta-analysis.

The main mechanism by which BBR improves IR is by inhibiting the function of mitochondria, accelerating the breakdown of glycogen, and activating the AMPK signaling pathway.^[21,22]^ Although BBR has been shown to be effective in T2DM with IR by multiple trials, its safety and efficacy are not systematically evaluated, so it is necessary to systematically evaluate the published RCTs to provide evidence-based medical evidence for their clinical use. The process of performing this systematic review, as shown in Fig. 2, includes identification of studies, selection of studies, data extraction and management, and data analysis.

Nevertheless, this review still has some limitations. First, there is a certain language bias in this study, which only includes Chinese and English. Second, due to the different doses of BBR, there may be significant heterogeneity here. Finally, small sample size tests may lead to a higher risk bias.

## Author contributions

**Data curation:** Yinshan Wang, Aihua Yan.

**Formal analysis:** Yinshan Wang, Bei Liu.

**Funding acquisition:** Yong Yan.

**Methodology:** Aihua Yan.

**Project administration:** Shanshan Li, Huimin Li.

**Software:** Bei Liu, Huimin Li.

**Supervision:** Yong Yan.

**Validation:** Yong Yan.

**Visualization:** Shanshan Li, Yong Yan.

**Writing – original draft:** Yinshan Wang, Yong Yan.

**Writing – review & editing:** Yinshan Wang, Yong Yan.

## References

[1] American Diabetes Association. Standards of medical care in diabetes-2014. Diabetes Care 2014;37 supp1:S14–80. 24357209 10.2337/dc14-S014

[2] Chen L Magliano DJ Zimmet PZ The worldwide epidemiology of type 2 diabetes mellitus-present and future perspectives. Nat Rev Endocrinol 2011;8:222–36. 10.1038/nrendo.2011.183 22064493

[3] Tessari P Cecchet D Cosma A Insulin resistance of amino acid and protein metabolism in type 2 diabetes. Clin Nutr 2011;30:267–72. 21492974 10.1016/j.clnu.2011.02.009

[4] Jaiswal M Divers J Pop-Busui R Response to Comment on Jaiswal et al. Prevalence of and Risk Factors for Diabetic Peripheral Neuropathy in Youth With Type 1 and Type 2 Diabetes: SEARCH for Diabetes in Youth Study. Diabetes Care 2018;41:e37. 29463674 10.2337/dci17-0058 PMC5829960

[5] Kahn SE Hull RL Utzschneider KM Mechanisms linking obesity to insulin resistance and type 2 diabetes. Nature 2006;444:840–6. 17167471 10.1038/nature05482

[6] Hidalgo B Irvin MR Sha J Epigenome-wide association study of fasting measures of glucose, insulin, and HOMA-IR in the Genetics of Lipid Lowering Drugs and Diet Network study. Diabetes 2014;63:801–7. 24170695 10.2337/db13-1100 PMC3968438

[7] Hwang YC Jeong IK Ahn KJ The uncarboxylated form of osteocalcin is associated with improved glucose tolerance and enhanced beta-cell function in middle-aged male subjects. Diabetes 2010;25:768–72. 10.1002/dmrr.1045 19877133

[8] Emoto M Nishizawa Y Maekawa K Homeostasis model assessment as a clinical index of insulin resistance in type 2 diabetic patients treated with sulfonylureas. Diabetes Care 1999;22:818–22. 10332688 10.2337/diacare.22.5.818

[9] Abbas SY Basyouni WM El-Bayouki KA Synthesis and evaluation of 1-substituted-biguanide derivatives as anti-diabetic agents for type II diabetes insulin resistant. Drug Res 2016;66:377–83. 10.1055/s-0042-107349 27191826

[10] Delgado H Lehmann T Bobbioniharsch E Acarbose improves indirectly both insulin resistance and secretion in obese type 2 diabetic patients. Diabetes Metab 2002;28:195–200. 12149599

[11] Suzuki M Takamisawa I Yoshimasa Y Association between insulin resistance and endothelial dysfunction in type 2 diabetes and the effects of pioglitazone. Diabetes Res Clin Pract 2007;76:12–7. 17007957 10.1016/j.diabres.2006.07.033

[12] Turner N Li JY Gosby A Berberine and its more biologically available derivative, dihydroberberine, inhibit mitochondrial respiratory complex I: a mechanism for the action of berberine to activate AMP-activated protein kinase and improve insulin action. Diabetes 2008;57:1414–8. 18285556 10.2337/db07-1552

[13] Zhao HL Sui Y Qiao CF Sustained antidiabetic effects of a berberine-containing Chinese herbal medicine through regulation of hepatic gene expression. Diabetes 2012;61:933–43. 22396199 10.2337/db11-1164 PMC3314348

[14] Martínez N White V Kurtz M Activation of the nuclear receptor PPARα regulates lipid metabolism in foetal liver from diabetic rats: implications in diabetes-induced foetal overgrowth. Diabetes 2011;27:35–46. 10.1002/dmrr.1151 21218506

[15] Wei XC Zhu LQ Wang CG Efficacy and safety of berberine in patients with type 2 diabetes mellitus: a meta-analysis. Chin Herbal Med 2015;7:344–53.

[16] Dai P Wang J Lin L Renoprotective effects of berberine as adjuvant therapy for hypertensive patients with type 2 diabetes mellitus: evaluation via biochemical markers and color Doppler ultrasonography. Exp Ther Med 2015;10:869–76. 26622407 10.3892/etm.2015.2585 PMC4533140

[17] De-Zeng T Yong-Xuan Z Xiao-Hua W Short term effects of berberine on T2DM resistance to external insulin. Chin J Exp Traditional Med Formulae 2010;16:174–5.

[18] Langer G Meerpohl JJ Perleth M GRADE guidelines: 12. Developing Summary of Findings tables—dichotomous outcomes. Z Evid Fortbild Qual Gesundhwes 2013;107:646–64. 24315336 10.1016/j.zefq.2013.10.034

[19] Bonora E Formentini G Calcaterra F HOMA-estimated insulin resistance is an independent predictor of cardiovascular disease in type 2 diabetic subjects: prospective data from the Verona Diabetes Complications Study. Diabetes Care 2002;25:1135–41. 12087010 10.2337/diacare.25.7.1135

[20] Jung CH Kim BY Kim CH Associations of serum fetuin-A levels with insulin resistance and vascular complications in patients with type 2 diabetes. Diab Vasc Dis Res 2013;10:459–67. 23811603 10.1177/1479164113490766

[21] Gomes AP Duarte FV Nunes P Berberine protects against high fat diet-induced dysfunction in muscle mitochondria by inducing SIRT1-dependent mitochondrial biogenesis. Biochim Biophys Acta 2012;1822:185–95. 22027215 10.1016/j.bbadis.2011.10.008 PMC3366688

[22] YuYZhaoYTengF Berberine improves cognitive deficiency and muscular dysfunction via activation of the AMPK/SIRT1/PGC-1a pathway in skeletal muscle from naturally aging rats. J Nutr Health Aging 2018;22:710–7.2980686010.1007/s12603-018-1015-7

